# Practical Treatise on Electricity in Gynecology

**Published:** 1891-12

**Authors:** 


Practical Treatise on Electricity in Gynecology. By Egbert H. Grandin, M. D., Chairman Section on Obstetrics and Gynecology, New York Academy of Medicine; Obstetric Surgeon, New York Maternity Hospital; Obstetrician New York Infant Asylum, etc., and Josephus H. Gunning, M. D., Instructor in Electro-Therapeutics, New York Post-Graduate Medical School and Hospital; Gynecologist to Riverview Rest for Women; Electro-Gynecologist North-Eastern Dispensary, etc. Illustrated. Octavo, 180 pages. Muslin. $2.00. New York: William Wood & Company. 1891.

In regard to the application of electricity to the treatment of disease, especially as to the propriety of its use in gynecology, there will ever be two opinions. The operating surgeon will naturally claim to do better justice to his patients through the medium of the knife, dealing with conditions that he can both see and feel with his educated eyes and finger-tips. On the other hand, the physician who has not the taste or skill to do successful surgical work will naturally turn to electricity to aid him in the management of his cases. Since we must meet conditions as they are and not fit a fact to a theory, it is well for us to have some of the best literature possible to serve us as a guide to the application of an unknown quantity, like electricity, to the treatment of known diseases such as are met with by the gynecologist.

The authors of the book before us are entitled to the credit of having prepared the best treatise that has appeared up to the present time in this country on the subject of its title. The section of the book given to general conditions and descriptions of apparatus comprising the first fifty-four pages, is a *résumé* of what is generally found in the books on electricity. This section is illustrated with the usual number of cuts of apparatus. The next chapter on the routine uses of electricity contains much valuable advice with reference to that branch of the subject, and will well repay careful reading. The remaining chapters on electrolysis, static electricity, and the treatment of malignant growths by the galvano-cautery, and electricity in obstetrics, are all well written, and should command attention by reason of the honesty and ability of the authors, no matter whether the reader finds himself agreeing with them on all points or not. The book is well printed and closes with an index, which, though somewhat meagre, is, perhaps, ample for all practical purposes. As we said at the beginning, taken all in all, this is the best book on the subject that has yet appeared from an American press.

